# Analysis of Bacterial Pathogens Causing Complicating HAP in Patients with Secondary Peritonitis

**DOI:** 10.3390/antibiotics12030527

**Published:** 2023-03-06

**Authors:** Josef Chudáček, Petr Špička, Milan Kolar, Martin Stašek, Štefan Kolcún, Dušan Klos, Kristýna Hricová, Patrik Mlynarcik, Vendula Pudová, Olga Klementová, Rostislav Horáček

**Affiliations:** 1 Department of Surgery I, Faculty of Medicine and Dentistry, Palacký University Olomouc, Hněvotínská 976/3, 77515 Olomouc, Czech Republic; 2 Department of Microbiology, Faculty of Medicine and Dentistry, Palacký University Olomouc, Hněvotínská 976/3, 77515 Olomouc, Czech Republic; 3 Department of Anesthesiology, Resuscitation and Intensive Care, University Hospital Olomouc, Zdravotníků 248/7, 77900 Olomouc, Czech Republic

**Keywords:** peritonitis, pneumonia, bacteria, etiology

## Abstract

Background: Diffuse peritonitis is an acute abdominal condition characterized by high mortality. The main treatment modality is surgery, requiring a subsequent prolonged hospital stay. These patients are, among other things, at risk of developing hospital-acquired pneumonia (HAP), which considerably worsens their treatment outcomes. This study aimed to extend the existing knowledge by providing more detailed microbiological characteristics of complicating HAP in patients with secondary peritonitis, including the identification of isolated bacterial pathogens and their potential sources. Methods: The 2015–2019 retrospective study comprised all patients with an intraoperatively confirmed diagnosis of secondary diffuse peritonitis who were classified in accordance with the quick Sepsis Related Organ Failure Assessment scoring system. Results: HAP developed in 15% of patients. The 90-day mortality rates were 53% and 24% in patients with and without HAP; respectively. The most frequent pathogens responsible for HAP were *Pseudomonas aeruginosa*, *Klebsiella pneumoniae*, *Escherichia coli*, *Enterobacter cloacae* complex and *Enterococcus faecalis*. Multidrug resistance to antibiotics was found in 38% of bacterial pathogens. Clonal spread of these bacterial pathogens among patients was not detected. Rather, the endogenous characteristic of HAP was confirmed. Conclusions: The initial antibiotic therapy of complicating HAP in patients with secondary peritonitis must be effective mainly against enterobacteria, including strains with the production of ESBL and AmpC beta-lactamases, *Pseudomonas aeruginosa* and *Enterococcus faecalis*. The study further highlighted the importance of monitoring the respiratory tract bacterial microflora in patients with secondary peritonitis. The results should be used for initial antibiotic treatment of complicating HAP instances.

## 1. Introduction

Diffuse peritonitis is an acute abdominal condition characterized by high mortality. Based on its developmental course, infectious peritonitis may be classified as primary, secondary or tertiary [1]. Secondary peritonitis is the most common form, accounting for up to 90% of all intraabdominal infections; in these cases, surgery is the treatment of choice [2]. The condition develops as a complication of another abdominal pathology or due to bacterial seeding of the peritoneal cavity following peritoneal perforation as the peritoneum becomes in direct contact with infectious agents [3]. The mainstay of therapy for peritonitis is early surgical intervention with the elimination of the infectious source, decontamination of the abdominal cavity and comprehensive intensive care for the patient, mostly requiring a stay in the intensive care unit [4,5,6].

Despite rapid advances in novel surgical techniques, such as negative pressure wound therapy (NPWT), targeted antimicrobial therapy and progress in intensive care medicine, peritonitis therapy is still challenging and remains associated with poor outcomes. This is evidenced by the relatively high mortality rates, which are reported to range from 10% to 20% [7,8]. As elderly individuals having multiple comorbidities are most frequently affected, most of them are then admitted to intensive care units. Their stays are relatively long, and they are at risk of complications that considerably worsen their outcomes [9]. One of the most common infectious complications in these patients is hospital-acquired pneumonia (HAP), accounting for 10–47% of nosocomial infections, with mortality rates reported at 20–60% [10,11]. HAP develops during a patient’s stay in the hospital and is defined as occurring 48 h or more after admission, but no later than 14 days from discharge. It may be further classified as early-onset HAP, developing 48–96 h post-admission, or late-onset HAP, occurring five or more days after admission [10,11]. Ventilator-associated pneumonia (VAP), a HAP subtype, is often observed in secondary peritonitis patients with shock and the need for mechanical ventilation [11,12,13,14]. Only few studies have investigated HAP in patients with diffuse peritonitis, stating that it develops in 10–29% of them [8,12,13,14]. The earliest study reported mortality to be as high as 66% [12]. Generally, a broad range of potential bacterial pathogens is implicated in the etiopathogenesis of HAP, in particular enterobacteria (most commonly *Klebsiella pneumoniae*), *Pseudomonas aeruginosa* and *Staphylococcus aureus*. On the other hand, coagulase-negative staphylococci and viridans streptococci are unlikely to cause the infection [10,15,16]. Enterococci, potentially causing late-onset HAP, account for 5% of all bacterial etiological agents, as stated by Herkel et al. [17]. In this case, the source is the upper gastrointestinal tract, with pneumonia developing as a result of reflux and subsequent microaspiration of gastric contents, as confirmed in a study carried out by Pudová et al. [18]. The only recent detailed analysis of bacterial pathogens responsible for complicating HAP in patients with secondary peritonitis was conducted by Heredia-Rodríguez et al., who identified strains of *Acinetobacter* spp., *Klebsiella* spp. and *Pseudomonas aeruginosa* as the most common agents [8].

Initial antibiotic therapy for HAP is seriously hampered by antimicrobial resistance, leading to inadequate antibiotic therapy and subsequently increases in both morbidity and mortality. The impact of the resistance of bacterial pathogens responsible for VAP based on antibiotic therapy was illustrated by Luna et al. [19]; they reported 38% mortality among adequately treated patients (i.e., with effective antibiotic therapy) and mortality reaching as high as 91% in the case of inadequate therapy (i.e., resistant pathogens). Similarly, Herkel et al. found a statistically significant difference in mortality between adequate and inadequate antibiotic therapy of VAP; adequately treated patients had a mortality of 27% as compared with 45% following inadequate therapy, with bacterial pathogens being resistant to initial antibiotic therapy [17]. The risk of death is also increased by the delayed initiation of proper antibiotic therapy, including an adequate dosage [11].

The role of HAP developing in patients with secondary peritonitis was described in a 2016 study by Heridia-Rodríguez et al. [8]; this study aimed to extend the existing knowledge by providing more detailed microbiological characteristics of complicating HAP in patients with secondary peritonitis, including the identification of isolated bacterial pathogens and their potential sources.

## 2. Material and Methods

### 2.1. Patients with Secondary Peritonitis

This retrospective study performed in the Department of Surgery I of the Olomouc University Hospital was started in January 2015, and data collection was completed in December 2019. The study included all (n = 274) patients with an intraoperatively confirmed diagnosis of secondary diffuse peritonitis. The cases were classified into four groups according to the type of effusion as follows: (1) serous, chemical or other peritonitis; (2) biliary peritonitis; (3) purulent peritonitis; and (4) stercoral peritonitis. Surgery was performed by an experienced surgeon skilled in the management of diffuse peritonitis. After the abdominal cavity was accessed using laparotomy or laparoscopy, effusion samples were collected for microbiology testing. This was followed by irrigation of the abdominal cavity and solving the cause of secondary diffuse peritonitis, most frequently by resection of the affected organ. Then, the abdominal cavity was primarily closed with continuous flow or passive drains left in place. Other options were the open abdomen technique with NPWT or vacuum-assisted closure or the use of non-woven fabrics for temporary closure of the abdominal cavity. With the open abdomen technique, surgical revision was needed after 24–48 h. Based on the obtained data, patients were classified in accordance with the quick Sepsis Related Organ Failure Assessment (qSOFA) scoring system, able to identify septic patients at high risk of morbidity and mortality.

### 2.2. Patients with HAP

In our group of patients with secondary diffuse peritonitis, we then identified patients with HAP. Patients meeting the following criteria were included in the HAP group: new or progressive infiltrates on chest X-ray at least 48 h after hospital admission plus at least two signs of respiratory tract infection, such as a fever of 38 °C or higher; purulent sputum; leukocytosis (white blood cell count [WBC] > 12 × 10^3^/mm^3^) or leukopenia (WBC < 4 × 10^3^/mm^3^); auscultatory findings indicative of lung inflammation; cough; and respiratory insufficiency (PaO_2_/FiO_2_ ratio < 300 mmHg). Present lung inflammation was confirmed via computed tomography or chest radiography.

### 2.3. Isolation and Identification of Bacterial Pathogens Causing HAP

In the case of invasive airway management with orotracheal or tracheostomy tubes, samples for microbiology culture tests were collected through the aspiration of secretions from the lower respiratory tract. From patients without airway management, their sputum was sampled. Clinical samples were inoculated using standard bacteriological loops onto blood agar and MacConkey agar (Trios, Czech Republic) and incubated for 24 h at 35 ± 1 °C. Isolated bacterial colonies were identified using standard microbiology testing with the MALDI-TOF MS system (Biotyper Microflex, Bruker Daltonics, MBT Compass reference library version 2022) [20]. Only the first isolated strain from each patient was included in this study. Etiological agents causing HAP were only identified based on positive respiratory secretion, sputum or blood cultures. Upper respiratory tract swabs were used for the standard monitoring of bacterial flora (at least once a week), and the results (present bacterial strains and their resistance to antimicrobials) were used to determine the pattern of initial antibiotic therapy in patients developing HAP. Coagulase-negative staphylococci, viridans streptococci and *Neisseria* spp. were not considered as causing HAP and were not included in the study. All isolated strains were stored in cryotubes at −80 °C (Cryobank B, ITEST, Hradec Králové, Czech Republic).

### 2.4. Determination of Resistance to Antibiotics

Resistance/susceptibility to antibiotics was determined using a standard microdilution method in accordance with the EUCAST criteria [21]. Reference bacterial strains (*Escherichia coli* ATCC 25922, *Pseudomonas aeruginosa* ATCC 27853, *Staphylococcus aureus* ATCC 29213, *Enterococcus faecalis* ATCC 29212) were used for quality control. The production of broad-spectrum beta-lactamases (ESBL, AmpC, KPC, MBL, OXA) was detected through phenotypic testing and confirmed via PCR detection of the relevant genes [22,23,24,25]. Strains of *Staphylococcus aureus* were tested for resistance to methicillin using a selective chromogenic medium (Colorex/TM/MRSA, Trios) and an immunochromatographic assay for the detection of PBP2a (PBP2a SA Culture Colony Test, Alere^TM^). Resistance of vancomycin-resistant enterococci was assessed through detection of the relevant *vanA* and *vanB* genes [26]. A HAP-causing bacterial pathogen was defined as multiresistant if acquired (i.e., not natural) resistance to at least one representative, of three or more antibiotic classes, was confirmed [27].

### 2.5. Genetic Analysis of Bacterial Pathogens

The identity of isolated bacterial pathogens was determined in species accounting for at least 10% of the isolates, that is, *Pseudomonas aeruginosa*, *Klebsiella pneumoniae*, *Escherichia coli* and *Enterococcus faecalis.* The *Enterobacter cloacae* complex included three strains of *Enterobacter cloacae* and two strains of *Enterobacter hormaechei*, with clonality not being determined. Additionally, the analysis comprised strains of the above species isolated from the upper respiratory tract and blood cultures in case the same species was also isolated from endotracheal secretions or sputum obtained from a patient with clinically confirmed HAP. The clonality of bacterial isolates was assessed with pulsed-field gel electrophoresis (PFGE). Bacterial DNA was isolated according to a PFGE protocol (*Enterococcus faecalis*) and a modified PFGE protocol with 3% sodium dodecyl sulfate and 3% sarcosine (*Klebsiella pneumoniae*, *Escherichia coli*, *Pseudomonas aeruginosa*) [28,29]. DNA was digested with the restriction enzymes *Xba*I (*Klebsiella pneumoniae*, *Escherichia coli)*, *Sma*I (*Enterococcus faecalis*) and *Spe*I (*Pseudomonas aeruginosa*) according to the manufacturer’s instructions. The obtained DNA fragments were separated via PFGE on a 1.2% agarose gel at 6 V.cm^−1^ and pulse times of 2–35 s for 24 h (*Klebsiella pneumoniae*, *Escherichia coli*, *Enterococcus faecalis*) or 5–60 s for 23 h (*Pseudomonas aeruginosa*). The gels were then stained with ethidium bromide. The banding patterns were analyzed with GelCompar II software (Applied Maths, Kortrijk, Belgium) using the Dice coefficient (1.0%) for comparing similarity and the unweighted pair group method with arithmetic means for cluster analysis. Restriction profiles reaching 100% similarity were considered identical.

### 2.6. Statistical Methods

In our study, we assessed a number of qualitative and quantitative variables. Quantitative variables are presented as the mean and standard deviation (SD), minimum, maximum and median. The Shapiro–Wilk test was used to check whether quantitative variables were normally distributed. Two independent samples were compared with the Mann–Whitney U test. For qualitative data, absolute and relative frequencies were calculated. Differences between groups were assessed with Fisher’s exact test. All tests were performed at a 0.05 level of significance. If the *p*-value was less than 0.05, the difference was considered statistically significant (marked with an asterisk). The statistical analyses were performed with IBM SPSS Statistics for Windows, Version 23.0. Armonk, NY, USA: IBM Corp.

## 3. Results

Between January 2015 and December 2019, a total of 274 patients underwent surgery for secondary diffuse peritonitis. Of these, forty (14.6%) patients developed HAP. The mean ages of patients with and without HAP were 68 and 64 years, respectively. In both groups, the most frequent cause was perforation of the descending colon, accounting for 32.5% and 27.7% of cases in the HAP and non-HAP groups, respectively. Primary closure of the abdominal cavity with only passive drains was the most common treatment approach used in both groups. Effusions were mostly purulent (HAP group, 50.0%; non-HAP group, 60.7%). There was a statistically significant difference in the median qSOFA score, which was higher (1) in patients with HAP compared to that in those without HAP (0) (*p* = 0.021). Additionally, statistically significant differences in mortality were observed, with higher rates in the HAP group than in the non-HAP group: 30-day mortality, 17 (42.5%) vs. 45 (19.2%; *p* = 0.002), and 90-day mortality, 21 (52.5%) vs. 55 (23.5%; *p* = 0.0004). The overall 30- and 90-day mortality rates, irrespective of HAP status, were 22.6% and 27.7%, respectively (Table 1).

### 3.1. Bacterial Pathogens Causing HAP

HAP was confirmed in 40 patients, with bacterial pathogens being identified based on positive endotracheal secretion or sputum cultures in 34 (85.0%) patients. In one patient, the etiological agent, *Pseudomonas aeruginosa*, was detected from both their endotracheal secretion and blood culture. Of the 34 patients with identified bacterial pathogens, nine (26.5%) had HAP of a polymicrobial etiology. Table 2 shows bacterial species most frequently causing complicating HAP in patients with peritonitis. Out of 45 bacterial pathogens isolated, 77.8% were Gram-negative bacteria, mostly enterobacteria and *Pseudomonas aeruginosa* (68.9%). Among Gram-positive bacteria (22.2% of isolated bacterial pathogens), strains of *Enterococcus faecalis* were most frequently (11.1%) detected. The etiological role of enterobacteria (a total of 22 isolated strains) was demonstrated in 21 patients (61.8% of those with a known etiology); non-fermenting Gram-negative rods (13 strains) caused HAP in 11 (32.4%) patients.

### 3.2. Resistance to Antibiotics

Of the 45 bacterial pathogens, 17 (37.8%) isolates may be characterized as multiresistant, namely five strains of Pseudomonas aeruginosa, four ESBL-positive (especially CTX-M) strains of Klebsiella pneumoniae, three ESBL-positive (CTX-M) strains of Escherichia coli, three AmpC-positive strains of Enterobacter cloacae complex, one OXA-positive (OXA-23) strain of Acinetobacter baumannii and one vancomycin-resistant strain of Enterococcus faecium (VanA phenotype). Antibiotic susceptibility/resistance of all isolated bacterial pathogens is shown in the Supplementary Materials (Tables S1–S3).

### 3.3. Clonality of Selected Bacterial Isolates

**Figure 1:**
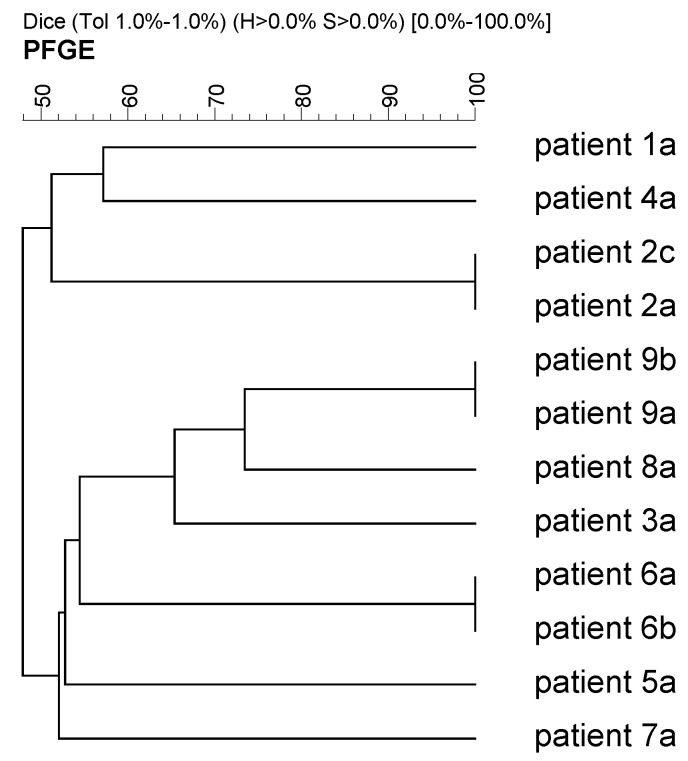
Dendrogram of Pseudomonas aeruginosa isolates from the respiratory tract and blood cultures. Horizontal axis—similarity of bacterial isolates (%); vertical axis—isolate number; letter a denotes isolates from endotracheal secretions, letter b denotes isolates from the upper respiratory tract and letter c denotes isolates from blood cultures.

**Figure 2:**
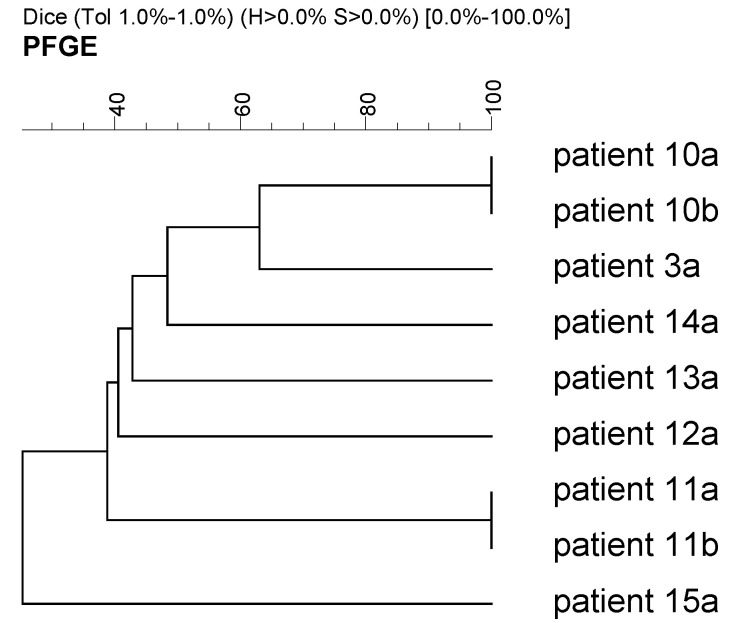
Dendrogram of Klebsiella pneumoniae isolates from the respiratory tract. Horizontal axis—similarity of bacterial isolates (%); vertical axis—isolate number; letter a denotes isolates from endotracheal secretion and letter b denotes isolates from the upper respiratory tract.

**Figure 3:**
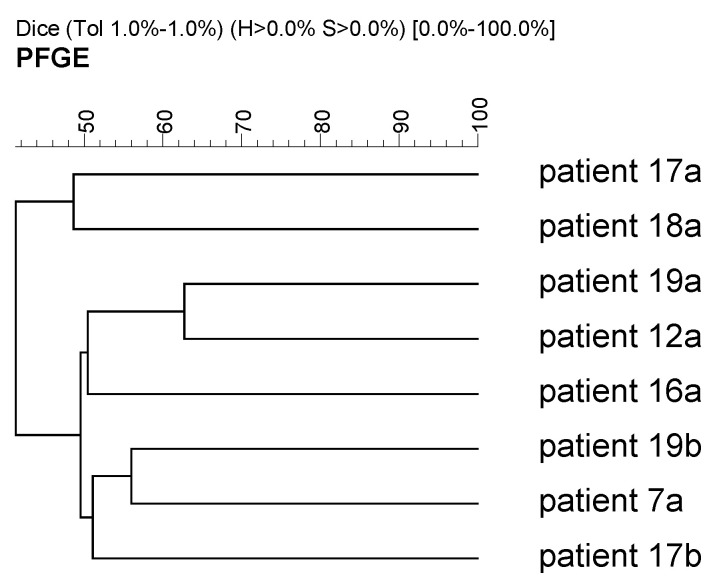
Dendrogram of Escherichia coli isolates from the respiratory tract. Horizontal axis—similarity of bacterial isolates (%). Vertical axis—isolate number; letter a denotes isolates from endotracheal secretion and letter b denotes isolates from the upper respiratory tract.

**Figure 4:**
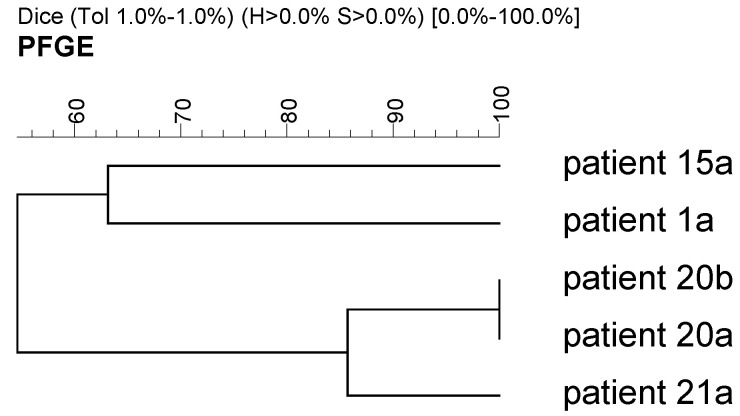
Dendrogram of Enterococcus faecalis isolates from the respiratory tract. Horizontal axis—similarity of bacterial isolates (%). Vertical axis—isolate number; letter a denotes isolates from endotracheal secretion and letter b denotes isolates from the upper respiratory tract.

Clonality analysis of Pseudomonas aeruginosa, Klebsiella pneumoniae, Escherichia coli and Enterococcus faecalis strains failed to reveal identical bacterial pathogens in patients (Figure 1, Figure 2, Figure 3 and Figure 4). This means that clonal, or horizontal, spread of these bacterial pathogens was not detected. However, in some patients, isolates were identical to those obtained from their upper respiratory tract prior to the development of HAP. Out of 21 patients with HAP caused by Pseudomonas aeruginosa, Klebsiella pneumoniae, Escherichia coli and Enterococcus faecalis strains, five (23.8%) were found to have had these bacterial pathogens in their upper respiratory tract before developing HAP. Molecular genetic analysis confirmed that the isolates were identical to those obtained from the lower respiratory tract after the onset of HAP (Figure 1, Figure 2 and Figure 4). Additionally, the Pseudomonas aeruginosa isolates obtained from both endotracheal secretion and blood cultures were confirmed to be identical (Figure 1).

## 4. Discussion

Nosocomial infections dramatically reduce the effectiveness of therapy and increase healthcare costs; HAP is one of the most common, accounting for nearly half of all nosocomial infections and increasing mortality to as much as 60% [10,11]. HAP poses a serious threat to, among others, patients with secondary peritonitis. Despite advances in surgical techniques, such as NPWT, antimicrobial therapy and supportive intensive care, peritonitis therapy remains arduous and its outcomes are unsatisfactory, as evidenced by mortality rates of 10–20% at best [7,8]. As most patients with secondary diffuse peritonitis are admitted to intensive care units and their condition is serious enough to require a long hospital stay, they are among those most at risk of developing HAP. Recently, only one study has been published on nosocomial bronchopneumonia in patients with secondary peritonitis [8]. The authors reported an overall incidence of acquired HAP of 10%, compared to 15% in the present study. In two pre-2000 studies, the incidence rates were as high as 30% [12,13].

The present study showed a statistically significant difference in qSOFA scores between patients with and without HAP. The former had higher qSOFA scores, and their increase was correlated with rising mortality [30]. Heredia-Rodríguez et al. confirmed that the SOFA score and duration of mechanical ventilation are independent factors for the development of HAP [8]. Higher mortality rates are predicted in patients with VAP and high SOFA scores [31]. Patients with HAP have mortality rates of 53–75% [12,13]. In the present study, statistically significant differences in mortality were observed between patient groups. Both 30- and 90-day mortality rates were higher in the HAP group (43% vs. 19% and 53% vs. 24%, respectively). The 90-day mortality rate (53%) was similar to the rate (48%) reported in the only recent study of patients with VAP [8]. In their studies, Riché et al. and Inui et al. explored risk factors for the treatment of patients with diffuse peritonitis; unfortunately, HAP was not mentioned [32,33]. The present study suggests that HAP significantly contributes to the mortality of patients with secondary peritonitis.

The present study found that in 85% of patients, bacteria causing HAP were present, most frequently Pseudomonas aeruginosa, Klebsiella pneumoniae, Escherichia coli, Enterobacter cloacae complex and Enterococcus faecalis. In 62% of patients with a confirmed etiology of HAP, enterobacteria were identified; non-fermenting Gram-negative rods caused HAP in 32% of patients. These results are consistent with data from a multicenter, prospective, observational study conducted in four centers in the Czech Republic between 2013 and 2015 that identified strains of Klebsiella pneumoniae, Pseudomonas aeruginosa, Escherichia coli, Enterobacter spp., Staphylococcus aureus and Burkholderia cepacia complex as pathogens most frequently responsible for HAP [17]. Similar to that in a study by Pudová et al., the etiological role of enterococci was confirmed in HAP patients included in the present study [18]. According to Heredia-Rodríguez et al., the most frequent bacterial pathogens that cause complicating HAP in patients with secondary peritonitis are Acinetobacter spp., Pseudomonas aeruginosa, Klebsiella pneumoniae and Staphylococcus aureus [8]. These results are concordant with the present study, the only exception being Acinetobacter spp., which accounted for only 4% of isolates. Genetic analysis of the most frequent bacterial pathogens suggest that their horizontal spread among patients could be ruled out. By contrast, five patients were found to have bacterial strains isolated from the upper respiratory tract before developing HAP that were identical to strains of the same species isolated from endotracheal secretions after HAP occurred. These results confirm the endogenous characteristic of HAP or the fact that the bacterial pathogens may originate from the upper respiratory tract microflora.

Among the isolated bacterial pathogens, 38% of isolates may be characterized as multiresistant. The most frequent mechanism of resistance was the production of broad-spectrum beta-lactamases (ESBL, AmpC), observed in 45% of enterobacteria. A vancomycin-resistant strain of Enterococcus faecium was identified as being of the VanA phenotype. Methicillin-resistant strains of Staphylococcus aureus were not detected.

Antibiotic treatment of HAP in patients with secondary peritonitis must be initiated immediately after the diagnosis is made. However, this no way implies that antibiotics may be randomly selected. Rather, the choice must be based on a qualified assumption that they will be sufficiently active against the most common bacterial pathogens. An essential prerequisite for initial antibiotic therapy is detailed knowledge of bacterial pathogens and their resistance to antibacterial agents in a particular epidemiological unit [34]. The present study findings suggest that initial antibiotic therapy for complicating HAP in secondary peritonitis patients must be effective mainly against strains of Pseudomonas aeruginosa, Klebsiella pneumoniae, Escherichia coli, Enterobacter cloacae complex and Enterococcus faecalis. At the same time, it is necessary to consider the presence of multidrug-resistant bacterial pathogens, particularly the production of ESBL and AmpC broad-spectrum beta-lactamases in the case of enterobacteria. An essential part of the adequate therapeutic approach is the timely collection (preferably before antibiotic therapy is initiated) of relevant clinical samples (endotracheal secretions, sputum, blood cultures) for microbiology testing. Depending on microbiology test results, the clinical status and inflammatory markers, antibiotic therapy should be modified or de-escalation should be started, that is targeted therapy based on determination of the etiological agent and its susceptibility to antibacterials. Such an approach increases the chances for successful therapy, reduces the risk of developing bacterial resistance and cuts healthcare costs.

The results of the study also indicate that bacterial monitoring is advisable, that is detecting potential bacterial pathogens in the upper respiratory tract (including identification of their susceptibility/resistance to antibiotics), even in the absence of clinical signs of HAP. If, subsequently, HAP develops, these results must be taken into account when deciding on the optimal pattern of initial antibiotic therapy.

## 5. Conclusions

Treatment outcomes for patients with secondary diffuse peritonitis are unfortunately still poor, with some of them developing HAP, which considerably exacerbates their already high mortality rates. The most frequently identified pathogens causing HAP were Pseudomonas aeruginosa, Klebsiella pneumoniae, Escherichia coli, Enterobacter cloacae complex and Enterococcus faecalis. Multidrug resistance to antibiotics was found in approximately one-third of bacterial pathogens. The study has underlined the importance of monitoring the respiratory tract bacterial microflora in patients with secondary peritonitis and detailed knowledge of bacterial pathogens and their resistance to antibacterial agents in a particular epidemiological unit before developing HAP. The results should be considered when planning initial antibiotic therapy, which is optimally already targeted.

The authors are aware of certain limitations of the study, namely its single-center and retrospective design.

## Figures and Tables

**Table 1:** Demographic and clinical characteristics of patients with peritonitis.

Variable	Patients with HAP (n = 40)	Patients without HAP (n = 234)	*p*-Value
age (mean years ± SD)	63.9 ± 18.2	61.6 ± 16.4	0.191 ^a^
male gender [n (%)]	25 (62.5)	129 (55.1)	0.491
comorbidities [n (%)]			
diabetes mellitus	8 (20.0)	46 (19.7)	1.000
hypertension	27 (67.5)	126 (53.8)	0.123
malignancies	17 (42.5)	88 (37.6)	0.599
chronic kidney disease	1 (2.5)	26 (11.1)	0.146
liver disease	6 (15.0)	15 (6.4)	0.098
lung disease	14 (35.0)	50 (21.4)	0.070
two or more serious conditions	29 (72.5)	140 (59.8)	0.159
peritonitis type—etiology [n (%)]			0.971
1 right colon	5 (12.5)	35 (15.0)	
2 left colon	13 (32.5)	65 (27.7)	
3 rectum	3 (7.5)	14 (6.0)	
4 small intestine	8 (20.0)	48 (20.5)	
5 biliary tract	3 (7.5)	26 (11.1)	
6 upper GIT	8 (20.0)	46 (19.7)	
peritonitis type—effusion [n (%)]			0.403
serous, chemical, other	5 (12.5)	30 (12.8)	
purulent	20 (50.0)	142 (60.7)	
stercoral	11 (27.5)	50 (21.4)	
biliary	4 (10.0)	12 (5.1)	
qSOFA [n (%)]			0.021 *^,a^
0	14 (35.0)	123 (52.6)	
1	15 (37.5)	76 (32.5)	
2	9 (22.5)	28 (12.0)	
3	2 (5.0)	7 (3.0)	
mortality [n (%)]			
30-day	17 (42.5)	45 (19.2)	0.002 **
90-day	21 (52.5)	55 (23.5)	0.0004 ***
overall morbidity [n(%)]	38 (95.0)	163 (69.7)	0.0004 ***
surgery type [n (%)]			0.774
NPWT	12 (30)	54 (23.1)	
abdominal cavity closure with fabric	3 (7.5)	15 (6.4)	
primary closure with continuous flow	11 (27.5)	73 (31.2)	
primary closure with passive drain	14 (35.0)	92 (39.3)	

* *p* < 0.05; ** *p* < 0.01; *** *p* < 0.001; ^a^ Mann–Whitney U test.

**Table 2:** Bacterial pathogens causing HAP.

Bacterial Species	No. of Isolates	Percentage
*Pseudomonas aeruginosa*	9	20.0
*Klebsiella pneumoniae*	7	15.6
*Escherichia coli*	6	13.3
*Enterococcus faecalis*	5	11.1
*Enterobacter cloacae* complex	5	11.1
*Staphylococcus aureus*	4	8.9
*Klebsiella aerogenes*	2	4.4
*Acinetobacter baumannii*	2	4.4
*Serratia marcescens*	1	2.2
*Stenotrophomonas maltophilia*	1	2.2
*Burkholderia cepacia* complex	1	2.2
*Enterococcus faecium*	1	2.2
*Providencia rettgeri*	1	2.2

Legend: Enterobacter cloacae complex—Enterobacter cloacae (3×), Enterobacter hormaechei (2×).

## Data Availability

The data is contained with the article and Supplementary Material.

## Supplementary Materials

The following supporting information can be downloaded at: https://www.mdpi.com/article/10.3390/antibiotics12030527/s1, Table S1: Antibiotic susceptibility/resistance of enterobacteria causing HAP in patients with secondary peritonitis; Table S2: Antibiotic susceptibility/resistance of nonfermenting Gram-negative bacteria causing HAP in patients with secondary peritonitis; Table S3: Antibiotic susceptibility/resistance of enterococci and Staphylococcus aureus causing HAP in patients with secondary peritonitis.
