# Supplementary material for: Analysis of Bacterial Pathogens Causing Complicating HAP in Patients with Secondary Peritonitis
Source: Antibiotics (Basel). 2023 Mar 6;12(3):527. doi: 10.3390/antibiotics12030527 (PMC10044605; doi:10.3390/antibiotics12030527)
Supplement: Supplementary file 1 [file antibiotics-12-00527-s001.zip › antibiotics-2244672-supplementary.pdf]

Supplementary Data

Table S1. Antibiotic susceptibility/resistance of enterobacteria causing HAP in patients with secondary peritonitis.

Bacterial strain	Antibiotics												
	AMP	AMS	PPT	CRX	CTX	CTZ	CPM	MER	GEN	AMI	CIP	COL	TIG
Klebsiella pneumoniae_1	R	R	R	R	R	R	R	S	R	S	R	S	S
Klebsiella pneumoniae_2	R	S	S	S	S	S	S	S	S	S	S	S	S
Klebsiella pneumoniae_3	R	R	R	R	R	R	R	S	R	S	R	S	S
Klebsiella pneumoniae_4	R	R	R	R	R	R	R	S	R	S	R	S	S
Klebsiella pneumoniae_5	R	S	S	S	S	S	S	S	R	S	S	S	S
Klebsiella pneumoniae_6	R	R	R	R	R	R	R	S	R	S	R	S	S
Klebsiella pneumoniae_7	R	S	S	S	S	S	S	S	S	S	S	S	S
Escherichia coli_1	R	R	R	R	R	R	R	S	R	S	R	S	S
Escherichia coli_2	S	S	S	S	S	S	S	S	S	S	S	S	S
Escherichia coli_3	R	R	R	R	R	R	R	S	R	S	R	S	S
Escherichia coli_4	R	S	S	S	S	S	S	S	S	S	S	S	S
Escherichia coli_5	S	S	S	S	S	S	S	S	S	S	S	S	S
Escherichia coli_6	R	R	R	R	R	R	R	S	R	S	R	S	S
Enterobacter cloacae_1	R	R	R	R	R	R	S	S	R	S	R	S	S
Enterobacter cloacae_2	R	R	R	R	R	R	S	S	R	S	R	S	S
Enterobacter cloacae_3	R	R	S	R	S	S	S	S	S	S	S	S	S
Enterobacter hormaechei_1	R	R	R	R	R	R	S	S	R	S	R	S	S
Enterobacter hormaechei_2	R	R	S	R	S	S	S	S	S	S	S	S	S
Klebsiella aerogenes_1	R	R	S	R	S	S	S	S	S	S	S	S	S
Klebsiella aerogenes_2	R	R	S	R	S	S	S	S	S	S	S	S	S
Serratia marcescens_1	R	R	S	R	S	S	S	S	S	S	S	R	R
Providencia rettgeri_1	R	R	S	R	S	S	S	S	S	S	S	R	R

Legend: S – susceptible, R – resistant, AMP – ampicillin, AMS – ampicillin/sulbactam, PPT – piperacillin/tazobactam, CRX – cefuroxime, CTX – cefotaxime, CTZ – ceftazidime, CPM – cefepime, MER – meropenem, GEN – gentamicin, AMI – amikacin, CIP – ciprofloxacin, COL – colistin, TIG – tigecycline

Table S2. Antibiotic susceptibility/resistance of nonfermenting Gram-negative bacteria causing HAP in patients with secondary peritonitis.

Bacterial strain	Antibiotics										
	AMS	PPT	CTZ	CPM	MER	GEN	AMI	CIP	COL	TIG	COT
Pseudomonas aeruginosa_1	R	R	R	R	R	R	S	R	S	R	R
Pseudomonas aeruginosa_2	R	S	S	S	S	S	S	S	S	R	R
Pseudomonas aeruginosa_3	R	R	R	R	R	R	S	R	S	R	R
Pseudomonas aeruginosa_4	R	R	R	R	R	R	S	R	S	R	R
Pseudomonas aeruginosa_5	R	R	R	R	R	R	S	R	S	R	R
Pseudomonas aeruginosa_6	R	S	S	S	S	S	S	R	S	R	R
Pseudomonas aeruginosa_7	R	R	S	S	S	R	S	S	S	R	R
Pseudomonas aeruginosa_8	R	R	S	S	S	S	S	S	S	R	R
Pseudomonas aeruginosa_9	R	R	R	R	R	R	S	R	S	R	R
Acinetobacter baumannii_1	S	S	S	S	S	S	S	S	S	S	R
Acinetobacter baumannii_2	R	R	R	R	S	R	S	R	S	S	R
Stenotrophomonas maltophilia_1	R	R	S	S	R	R	R	R	S	S	S
Burkholderia cepacia complex_1	R	R	S	S	S	R	R	R	R	S	S

Legend: S – susceptible, R – resistant, AMS – ampicillin/sulbactam, PPT – piperacillin/tazobactam, CTZ – ceftazidime, CPM – cefepime, MER – meropenem, GEN – gentamicin, AMI – amikacin, CIP – ciprofloxacin, COL – colistin, TIG – tigecycline, COT – cotrimoxazole

Table S3. Antibiotic susceptibility/resistance of enterococci and *Staphylococcus aureus* causing HAP in patients with secondary peritonitis.

Bacterial strain	Antibiotics								
	AMP	OXA	ERY	CLI	VAN	TEI	TIG	LNZ	COT
Enterococcus faecalis_1	S	NT	NT	NT	S	S	S	S	NT
Enterococcus faecalis_2	S	NT	NT	NT	S	S	S	S	NT
Enterococcus faecalis_3	S	NT	NT	NT	S	S	S	S	NT
Enterococcus faecalis_4	S	NT	NT	NT	S	S	S	S	NT
Enterococcus faecalis_5	S	NT	NT	NT	S	S	S	S	NT
Enterococcus faecium_1	R	NT	NT	NT	R	R	S	S	NT
Staphylococcus aureus_1	NT	S	S	S	S	S	S	S	S
Staphylococcus aureus_2	NT	S	S	S	S	S	S	S	S
Staphylococcus aureus_3	NT	S	S	S	S	S	S	S	S
Staphylococcus aureus_4	NT	S	S	S	S	S	S	S	S

Legend: S – susceptible, R – resistant, AMP – ampicillin, OXA – oxacillin, ERY – erythromycin, CLI – clindamycin, VAN – vancomycin, TEI – teicoplanin, TIG – tigecycline, LNZ – linezolid, COT – cotrimoxazole, NT – not tested
